# De Novo Blister Aneurysm Formation in 16 Days Associated with Mucorales Fungi

**DOI:** 10.7759/cureus.5301

**Published:** 2019-08-01

**Authors:** Harjot Thind, Ben Waldau

**Affiliations:** 1 Neurological Surgery, University of California Davis Medical Center, Sacramento, USA; 2 Neurological Surgery, University of California, Davis Medical Center, Sacramento, USA

**Keywords:** blister aneurysm, mucorales fungi, subarachnoid hemorrhage

## Abstract

Although blister aneurysms represent a small percentage of all intracranial aneurysms, they are generally considered to be a more morbid and challenging entity than the more common saccular intracranial aneurysms. Despite this, the etiology of blister aneurysms is still unknown, though there are several theories. We present the case of a 54-year-old man who initially presented with vision loss and normal intracranial computed tomography angiography imaging. Only 16 days thereafter, he underwent rapidly progressive clinical decline, which was found to be due to the development and rupture of a de novo supraclinoidal blister aneurysm. Autopsy results showed fungal infection of the arterial wall by Mucorales fungi at the site of the aneurysm. Our case report supports the theory that blister aneurysms can be caused by fungal infection of the wall of the internal carotid artery.

## Introduction

Blister aneurysms represent less than one percent of all intracranial aneurysm [1-2]. They are a separate and distinct entity from other intracranial aneurysms such as saccular and mycotic aneurysms. Various specific definitions can be found in the literature, but it is generally agreed upon that blister aneurysms occur at non-branching sites of proximal intracranial arteries and have a conical shape with a wide neck [1]. These aneurysms are also thought to be more morbid than saccular aneurysms as they have a higher re-rupture risk as well as increased risk for operative rupture during craniotomy and ischemic event [3]. These aneurysms are most commonly found on the dorsal surface of the supraclinoidal internal carotid artery (ICA) [4]. Although this entity has been described in the literature, the exact cause of blister aneurysms is yet unknown. A previous case report points towards an infectious etiology of blister aneurysms [5].

We present the development of a de novo supraclinoidal blister aneurysm in the setting of an autopsy proven fungal infection of the vessel wall by Mucorales fungi. Our case report supports the theory that blister aneurysms can be caused by fungal infection of the wall of the ICA.

## Case presentation

**Figure 1:**
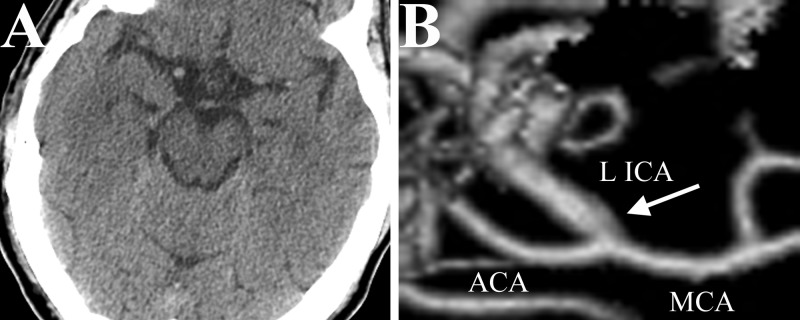
Baseline normal imaging studies A) Axial computed tomography (CT) of the brain without contrast showing no acute hemorrhage in basal cisterns. B) 3Dimensional reconstruction of CT angiography of the neck showing normal intracranial vessel anatomy, *white arrow* indicates left supraclinoidal internal carotid artery without evidence of aneurysm. ACA:* *anterior cerebral artery; LICA: left internal carotid artery; MCA: middle cerebral artery*.*

A 54-year-old right-handed gentleman, with a past medical history of poorly controlled type II diabetes mellitus, hepatitis C, and polysubstance abuse (cocaine and methamphetamine), initially presented to the emergency department with 24 hours of acute vision loss in his left eye as well as retro orbital pain. Physical exam was significant only for limited extraoccular motility due to left eye pain and relative afferent pupillary defect in the left eye with no light perception. He was admitted to the hospital and full workup, including magnetic resonance imaging (MRI) with and without contrast of the brain, orbit, face, and neck, computed tomography (CT) of the head (Figure 1A), computed tomography angiography (CTA) of the neck (Figure 1B), transthoracic echocardiogram, human immunodeficiency virus panel, and temporal artery biopsy, was negative for acute pathology. Pertinent abnormal values were hemoglobin A1C percentage of 10%, elevated erythrocyte sedimentation rate, elevated c-reactive protein, urine toxicology screen positive for methamphetamines, presence of HLA-B27 antigen, as well as the presence of antinuclear antibodies in a nucleolar pattern consistent with scleroderma, lupus, polymyositis, or other connective tissue disease. After a trial of steroids did not provide significant improvement in his symptoms, the working diagnosis for the patient’s vision loss was posterior ischemic optic neuropathy. He was discharged home after eight days in the hospital.

**Figure 2:**
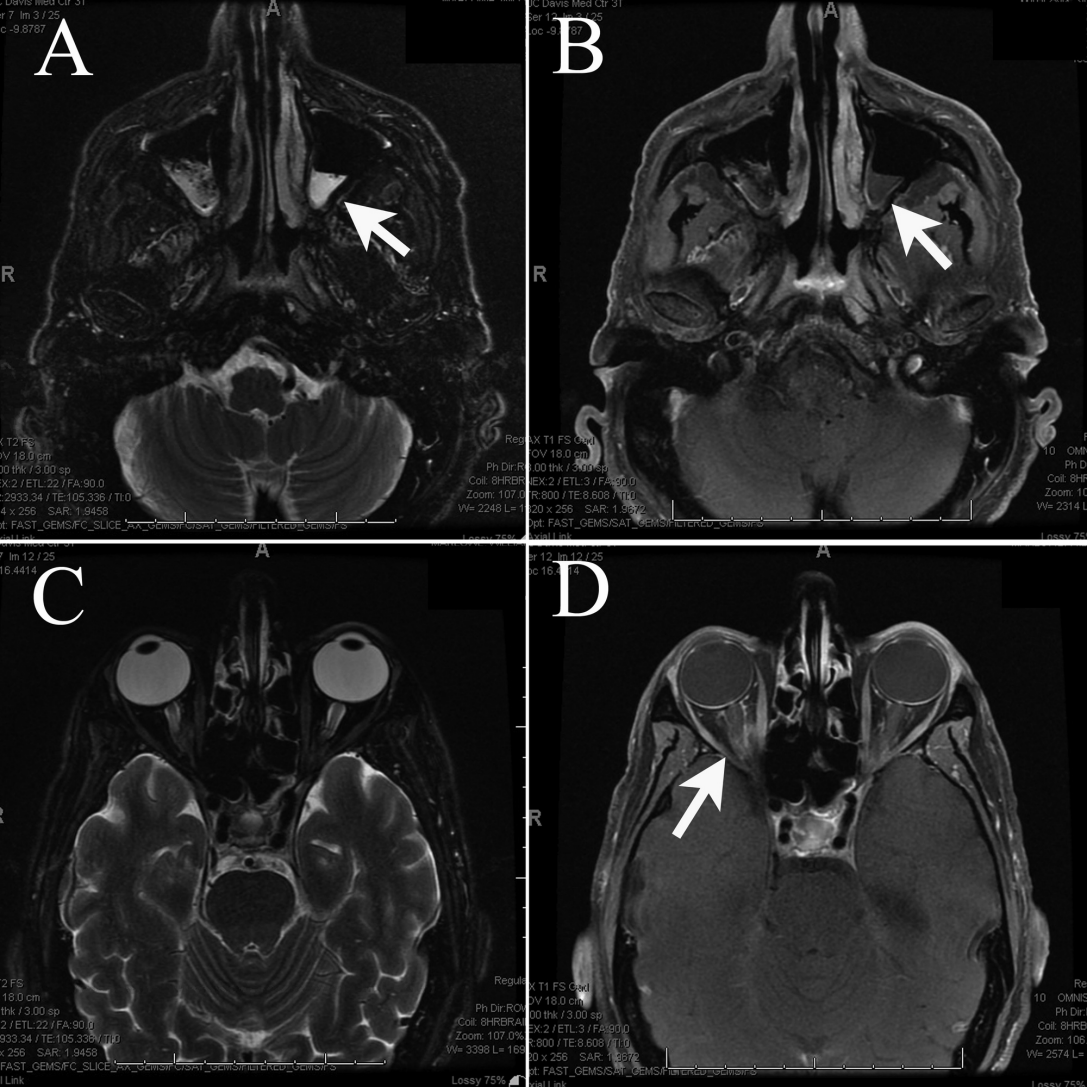
Magnetic resonance imaging (MRI) of the orbit/face/neck A) Axial T2 MRI and B) Axial T1 post-contrast MRI showing mild ethmoidal and maxillary sinus mucosal disease with fluid/air levels, *white arrows* indicate fluid/air levels in maxillary sinus. C) Axial T2 MRI and D) Axial T1 post-contrast MRI showing bilateral optic neuritis with suggestion of a more acute process on the right, *white arrow* indicates contrast enhancement on right optic nerve.

Two days after discharge from the hospital and 10 days after his initial presentation, the patient presented to the emergency department with acute right (contralateral to initial presentation) eye vision loss for the past 24 hours. Physical exam was significant for new right eye vision loss (no light perception) and left abducens nerve palsy with pallor and grade two edema of the left optic disc. Non-contrast CT of the head at that time showed fluid levels in bilateral maxillary sinuses but otherwise was negative for acute intracranial pathology. Formal MRI of the orbit/face/neck was completed on hospital day 3 and showed mild ethmoid and maxillary sinus mucosal disease with fluid/air levels (Figure 2A and 2B) and findings compatible with bilateral optic neuritis with suggestion of a more acute process on the right (Figure 2C and 2D). Steroid therapy was initiated. Lumbar puncture was performed on hospital day 4 which revealed elevated glucose but was otherwise within normal limits with negative culture results. Endoscopic sinus surgery, along with multiple bone biopsies, was performed on hospital day 6. Postoperative non-contrast head CT did not reveal any acute abnormalities.

**Figure 3:**
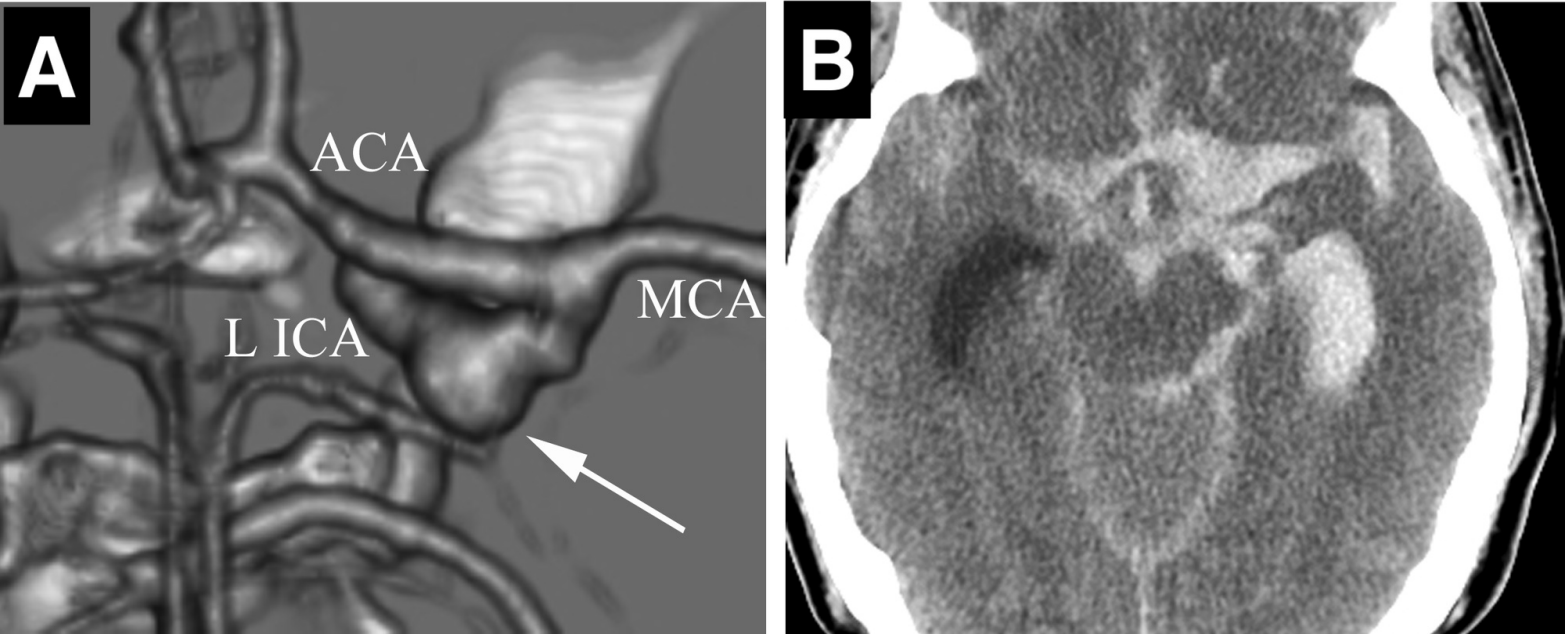
Repeat Imaging Studies A) 3Dimensional reconstruction of computed tomography (CT) angiography of the brain showing de novo blister aneurysm formation in the left supraclinoidal ICA (*arrow)*. B) Axial CT of the brain showing extensive subarachnoid hemorrhage from blister aneurysm rupture; *ACA- anterior cerebral artery, L ICA- left internal carotid artery, MCA- middle cerebral artery.*

The patient had left eye pain postoperatively however remained neurologically stable until two days postoperatively when he had an acute change in his neurologic status with decreased level of consciousness and right-sided weakness. The patient was emergently transferred to the intensive care unit and intubated for airway control. Emergent CTA revealed a new left 6.5mm supraclinoidal internal carotid artery blister aneurysm as shown in Figure 3A without evidence of hemorrhage. However, MRI was done afterwards which showed diffuse T2 hyperintensities within the major subarachnoid spaces of the skull base as well as bilateral frontal and temporal sulci concerning for acute subarachnoid hemorrhage and a left anterior choroidal artery (origin of which is in the length of the aneurysm). Lumbar puncture was performed due to concern for basilar meningitis and showed an opening pressure of 55 cmH2O and gross blood. Repeat CT head showed extensive subarachnoid hemorrhage (Figure 3B) mildly progressed from previous MRI with moderate ventriculomegaly and diffuse cerebral edema with inferior tonsillar herniation. Discussion with family was held and decision was made to stop aggressive medical care. The patient was transitioned to comfort measures only and expired the next day.

Pathology results from the patient’s left sphenoid sinus biopsy showed fragmented fungal hyphae associated with eosinophilic and necrotic debris. Autopsy revealed extensive broad, irregularly shaped, non-septate hyphae with right-angle branching and oval conidia in the necrotic and hemorrhagic brain tissue, consistent with the morphological features of the Mucorales species and confirmed by special staining. These were seen admixed with and invading cranial nerves, nervous tissue, and blood vessel walls. The left distal ICA showed broad nonseptate hyphae as well. In the necrotic brain tissue and nerves with hemorrhage, there were Mucorales hyphae and conidia that were tightly adherent to and invading the blood vessel walls, which were thickened with eosinophilic material.

## Discussion

To the best of our knowledge, this is the first report of a radiographically documented de novo blister aneurysm formation of the supraclinoidal internal carotid artery due to fungal Mucorales species.

The pathophysiology of blister aneurysms still remains largely unknown, although there are two general theories on their formation. One thought is that they are due to dissections, as dissection has been seen in 40% to 89% of patients with a blister aneurysm [3]. In this theory, they are considered “pseudoaneurysms” as histological evidence has shown a tenuous fibrin layer in the aneurysmal segment [3,6]. However, other histopathologic studies indicate that arteriosclerosis may be the causative factor. In the study by Regelsberger et al., autopsy studies showed arteriosclerotic segments and different areas where elastic fibers had been lost [7]. In the study by Inshikawa et al., pathologic investigations showed that at the rupture point, there was “eccentric arteriosclerosis” of the wall with hypertrophied intimal layer with no internal elastic lamina and no media layer, and this disappearance of the media was seen acutely at the boundary between the sclerotic and normal portions of the vessel [8]. More interestingly, their pathology did not show any collagenous tissue (as usually seen in saccular aneurysms) and no dissection [8]. The deduction is that arteriosclerosis is the main factor in degeneration of the internal elastic lamina, thereby leaving the vessel vulnerable to develop a blister aneurysm [8-9]. The factor which is likely the second hit to actually cause the blister aneurysm is hypertension and the associated increased hemodynamic stress that occurs in this pathologic state [9-10]. This increased stress in a vulnerable segment can then cause the blister aneurysm and likely explains the unusual location of blister aneurysms as opposed to saccular aneurysms.

Although there are two prevailing theories on blister aneurysm formation, an infectious source of blister aneurysms has been proposed once previously. In their report, Ogawa et al. demonstrate Aspergillus as the cause of a de novo blister aneurysm [5]. As opposed to dissection, they demonstrated pathologic “destruction of the internal elastic lamina and media” in the aneurysm wall [5]. They also did note atherosclerosis in the ICA with no evidence of dissection. This supports the theory of a two-hit phenomenon for blister aneurysmal formation, and that fungal infection can cause one and/or both of those factors.

We add to this theory by demonstrating a de novo blister aneurysm caused by the Mucorales fungal species. Mucor is considered to be the most aggressive fungus in the central nervous system [11]. It is also potent in that it has a predilection for attacking the internal elastic lamina of arteries, and by reproducing in this layer, it separates the media from the internal elastic lamina [12-14]. Mucor produces proteinases that cleave the protein elastin [13,15]. Elastin is a large component of the internal elastic lamina, and this thus explains the effectiveness of the angioinvasion of Mucor. As Mucor invades the arteries and destroys the internal elastic laminar segment in vessels, it creates a weakened vessel wall segment. This then leaves the artery at high risk of aneurysm formation and likely explains the pathophysiology of blister aneurysm formation by the fungus Mucorales.

## Conclusions

Given the above information, we postulate that the angioinvasive and destructive nature of Mucorales fungi may cause a subset of blister aneurysms.
